# Sex and risk of hyperprogressive disease in cancer patients receiving immune checkpoint inhibitors: a systematic review and meta-analysis

**DOI:** 10.3389/fimmu.2026.1860831

**Published:** 2026-09-16

**Authors:** Jia Zhu, Changling Tu

**Affiliations:** Department of Geriatric Oncology, Yunnan Cancer Hospital, The Third Affiliated Hospital of Kunming Medical University, Peking University Cancer Hospital Yunnan, Kunming, China

**Keywords:** hyperprogressive disease, immune checkpoint inhibitors, immunotherapy, meta-analysis, sex

## Abstract

**Background:**

Hyperprogressive disease (HPD) refers to a phenomenon characterized by a significant acceleration in tumor growth during treatment with immune-checkpoint inhibitors (ICIs), far exceeding the pre-treatment growth rate, leading to rapid deterioration in the patient’s condition. HPD typically occurs within the first two months of treatment and often results in early mortality. While sex differences in immune responses have been extensively studied, the relationship between sex and HPD risk remains unclear.

**Methods:**

We systematically searched MEDLINE (PubMed), Embase, and Scopus databases to identify studies reporting HPD in patients treated with ICIs, with available gender-stratified data. The search encompassed all records from database inception until 30 June 2025. Meta-analyses were performed using RevMan 5.4.1 software, with pooled odds ratios and 95% confidence intervals calculated under either fixed-effects or random-effects model, depending on heterogeneity. The primary objective was to evaluate the association between biological sex and the risk of HPD. The research protocol has been registered on the PROSPERO platform (CRD420261324007).

**Results:**

This meta-analysis included 20 studies involving 2,291 patients (1,612 male patients and 679 female patients), all of which were retrospective. The overall incidence of HPD was 18.67% in male patients and 18.26% in female patients. Pooled analysis revealed no statistically significant association between biological sex and the risk of HPD development (*I*^2^ = 27%; *p* = 0.13).

**Conclusion:**

Our findings demonstrate that sex should not be considered a determinative factor for HPD risk in patients receiving ICIs.

## 1 Introduction

The emergence of immune checkpoint inhibitors (ICIs) has completely transformed the treatment landscape of advanced cancer, significantly improving the prognosis of some patients (1, 2). However, the efficacy of immunotherapy varies considerably across patient populations, and among the factors that may influence treatment outcomes, sex has emerged as a potential biological determinant (3). Sex differences in immune responses are well-documented. Women generally exhibit stronger innate and adaptive immune responses, while men are more susceptible to infections and malignancies (3, 4). In oncology, men not only have a higher cancer incidence but also a higher cancer mortality rate across multiple tumor types, including liver, bladder, esophageal, and head and neck cancers (5, 6). These disparities are largely attributable to genetic and hormonal factors. Notably, women possess two X chromosomes, which harbor numerous immune-related genes—including FOXP3, TLR7, CD40L, and CXCR3—that can escape X-chromosome inactivation, leading to higher expression levels and more robust immune activation in women (7). Concurrently, sex hormones such as estrogen and testosterone differentially modulate immune cell function, with estrogen generally promoting humoral immunity and testosterone exerting immunosuppressive effects (3). These molecular differences collectively underpin the observed sex disparities in immune competence and may have significant implications for the efficacy and safety of cancer immunotherapy.

Hyperprogressive disease (HPD) is a rare but highly aggressive pattern of tumor progression following ICIs, characterized by accelerated tumor growth and rapid clinical deterioration, often occurring within the first 2 months of treatment (8). The mechanisms underlying HPD remain incompletely understood, but emerging evidence suggests that abnormal remodeling of the immune microenvironment plays a central role (9, 10). Several potential pathways have been implicated: accelerated T-cell exhaustion kinetics driven by dysfunctional reinvigoration of exhausted T cells, expansion and recruitment of myeloid-derived suppressor cells (MDSCs) and regulatory T cells (Tregs) in response to programmed cell death protein 1 (PD-1)/programmed death-ligand 1 (PD-L1) blockade, and the upregulation of alternative immune checkpoints that suppress antitumor immunity (9, 11, 12). Given the established sex differences in immune cell composition, cytokine profiles, and the expression of X chromosome-linked immunoregulatory genes (e.g., FOXP3 and TLR7), it is biologically plausible that sex may influence the risk of HPD by modulating these microenvironmental processes.

Clinical evidence regarding the association between sex and HPD risk, however, remains conflicting. Kanjanapan et al. (13) reported a higher incidence of HPD among female patients (*p* = 0.01) in early-phase immunotherapy trials. Similarly, Saâda-Bouzid et al. (14) observed that gender was associated with HPD occurrence in patients with recurrent/metastatic head and neck squamous cell carcinoma. In contrast, several recent studies, including those by Petrova et al. (15), Aoki et al. (16), and Kim et al. (17), have failed to identify a significant sex difference. These divergent findings highlight the need for a systematic synthesis of the available evidence to clarify whether sex truly represents an independent risk factor for HPD.

## 2 Methods

This study was conducted according to the Preferred Reporting Items for Systematic Reviews and Meta-analyses (PRISMA) guidelines and registered in the International Prospective Register of Systematic Reviews, with the registration number CRD420261324007. This systematic review adheres to the PRISMA 2020 statement. The completed PRISMA checklist is provided as **Supplementary Material S1**.

### 2.1 Retrieval methods

For the current analysis, two researchers (ZJ and TCL) extracted data from MEDLINE (PubMed), Embase, and Scopus up to 30 June 2025, using the following search terms: immunotherapy, programmed death-ligand 1, PD-L1, cytotoxic T-lymphocyte-associated protein 4, CTLA-4, ipilimumab, tremelimumab, nivolumab, pembrolizumab, immune checkpoint inhibitor, hyperprogression, and hyperprogressive disease, and HPD. All duplicate records were excluded. We reviewed and excluded references from reviews, commentaries, editorials, included studies, and conference publications to ensure the accuracy of the included studies.

### 2.2 Inclusion and exclusion criteria

#### 2.2.1 Inclusion criteria

The inclusion criteria were as follows: (1) prospective or retrospective studies reporting the characteristics of patients who developed HPD during immunotherapy, regardless of tumor type; (2) all patients were diagnosed with malignancy via biopsy; and (3) numerical data on sex and HPD incidence were available.

#### 2.2.2 Exclusion

The exclusion criteria included: (1) duplicate studies, reviews, case reports, letters, reference abstracts, or studies for which full-text English articles were unavailable; (2) nonhuman studies, such as *in vitro* or animal studies; and (3) studies that did not provide numerical data on gender and HPD incidence.

### 2.3 Study selection and quality assessment

The literature retrieval was conducted independently by two authors. Any discrepancies were resolved through discussion with a third author until a consensus was reached.

Quality assessment was conducted using the Newcastle–Ottawa Scale (NOS), which evaluates study design based on eight criteria: selection of the study population, comparability of groups, and exposure. Only those with a score of ≥ 7 were included in this analysis.

### 2.4 Data extraction

Study characteristics were extracted, including the first author, publication year, study design, treatment type (PD-1, PD-L1, or cytotoxic T-lymphocyte antigen 4 (CTLA-4)), total number of HPD cases, and the number of male and female participants.

### 2.5 Statistical analysis

The pooled odds ratios (ORs) and 95% confidence intervals (CIs) were calculated using either random- or fixed-effects models to assess the association between sex and HPD risk. Heterogeneity across studies was evaluated using the *χ*^2^-based *Q* test, and the *I*^2^ statistic was computed to quantify the percentage of total variability attributable to study heterogeneity. When heterogeneity is significant (*I*^2^ ≥ 50% or *p* < 0.10), a random-effects model is selected. Publication bias was assessed through funnel plot visualization supplemented by Begg’s test. In addition, each study was excluded from the meta-analysis for sensitivity analyses. To further reduce heterogeneity among studies and enhance the reliability of the results, stratified analyses were conducted. When stratified analysis failed to eliminate heterogeneity in the pooled effect size, individual studies were sequentially excluded, and the effect size was recalculated to identify studies contributing significantly to heterogeneity. All statistical tests were two-tailed, with a significance threshold of *p* < 0.05, and were implemented using RevMan 5.4.1.

## 3 Results

### 3.1 Characteristics of included studies

#### 3.1.1 General characteristics of the studies

The search was updated to 30 June 2025, and a total of 835 articles were included. After full-text screening, 39 potentially relevant articles were identified. Ultimately, six studies (13, 15–19) met the inclusion criteria for both abstract and full-text review.

Additionally, 14 trials included in the meta-analysis by Zhao et al. (17) were also included. Therefore, a total of 20 retrospective studies were included in this meta-analysis (**Figure 1**), with publication dates ranging from 2019 to 2025. The sample sizes per trial ranged from 34 to 263. Among all the included patients, 15 studies were from Asian countries, including five from China, five from South Korea, and five from Japan; the other five studies were from non-Asian countries, including one from Canada, one from the Netherlands, one from Australia, one from Italy, and one from Bulgaria. All included studies were in English and had an NOS score of 7 or higher (**Table 1**).

**Figure 1:**
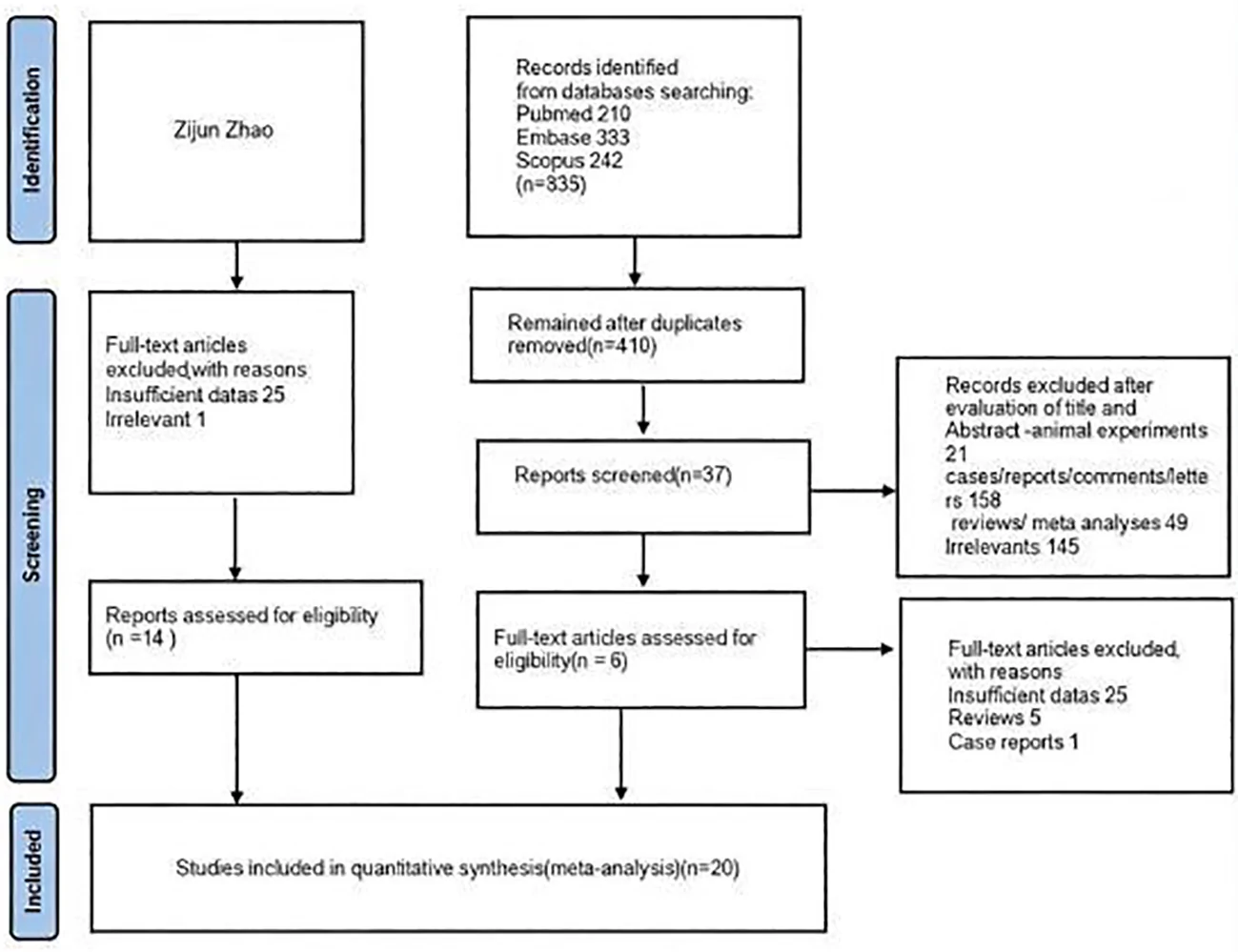
Details of the literature search and the study-selection processes.

**Table 1:** Fundamental characteristics of the included studies.

Author	Year	*N* (male/female)	Study design	Country	Underlying malignancy	Treatment	Definition of HPD	HPD male	HPD female	Quality assessment of NOS	Incidence of HPD
Aoki et al. (18)	2019	34 (26/8)	R	Japan	Gastric cancer	Nivolumab	TGRpost/TGRpre ≥ 2	8	2	High	10/34 (29.4%)
Aoki et al. (16)	2023	147 (108/39)	R	Japan	Gastric cancer	Nivolumab	A more or less two fold increase in the tumor growth rate of measurable lesions	33	8	High	41/147(27.9%)
Castello et al. (19)	2020	46 (32/14)	R	Italy	NSCLC	Nivolumab, pembrolizumab, atezolizumab	PD on the first CT scan according to RECIST 1.1 (TGRpost-TGRpre)/TGRpre ≥ 50%	8	6	High	14/46 (30.4%)
Chen et al. (20)	2023	107 (73/34)	R	China	Pancreatic cancer	PD(L)1	TGRpost−TGRpreb > 50%	8	2	High	10/107 (9.3%)
Hagi et al. (21)	2020	136 (107/29)	R	Japan	Gastric cancer	Nivolumab	> 50% increase in the sum of the longest diameters of the target lesions TGKpost/TGKpre ≥ 2	25	5	High	30/136 (22.1%)
Jin et al. (22)	2021	41 (28/13)	R	China	NPC	Anti-PD-1 mAb	(1) The acceleration of tumor cell proliferation exceeding two fold based on three time points (2) TTF ≤ 2 months	13	3	High	16/41 (39%)
Kanjanapan et al. (19)	2019	182 (99/83)	R	Canada	Multiple cancers	Anti-PD-1 mAb, anti-PD-L1 mAb, anti-CTLA-4 mAb	RECIST 1.1 progression at the first on treatment scan with a 2-fold or greater increase in TGR between the reference and experimental periods.	2	10	High	12/182 (6.6%)
Kanjanapan et al. (23)	2023	87 (51/36)	R	Australia	Lung cancer and melanoma	PD(L)1, CTLA-4	An increase of ≥ 40% in the sum of the target lesions compared with baseline and/or an increase of ≥ 20% in the sum of the target lesions compared with baseline plus new lesions in at least two different organs.	5	6	High	11/87 (12.6%)
Kim CG et al. (24)	2021	263 (191/72)	R	Korea	NSCLC	PD(L)1	Defined as TGRpost/TGRpreb ≥2 and TGKpost/TGKprec ≥ 2 according to the RECIST 1.1 and TTF <2 mont	34	20	High	54/263 (20.5%)
Kim J, et al. (17)	2022	219 (169/50)	R	Korea	NSCLC	Nivolumab, pembrolizumab, atezolizumab, durvalumab	TGKpost = (Vafter – Vbase/Tafter − Tbase) TGKpost/TGKpre ≥ 2 TTF ≤ 2 months	24	11	High	35/219 (16.0%)
Kim S. H, et al. (25)	2020	40 (24/16)	R	Korea	NSCLC	Nivolumab	HPD according to RECIST 1.1 at the first evaluation TGRpost/TGRpre ≥ 2	8	8	high	16/40 (40%)
Kim Y. et al. (26)	2019	137 (94/41)	R	Korea	NSCLC	Nivolumab, pembrolizumab, atezolizumab, durvalumab, avelumab	TTF < 2 months TGKpost/TGKpre ≥ 2 > 50% increase in the sum of the total measured tumor burden	34	14	High	48/154 (35.6%)
Lu et al. (27)	2019	56 (39/17)	R	China	GI tract cancer	Anti-PD-1 mAb, anti-PD-L1 mAb, anti-CTLA-4 mAb	NA	3	2	High	5/56 (8.9%)
Li Y, et al. (28)	2024	105 (22/83)	R	China	NSCLC	PD(L)1, CTLA-4	> 50% increase in the sum of the longest diameters of the target lesions TGKpost/TGKpre ≥ 2	33	11	High	44/105 (41.0%)
Petrova et al. (15)	2020	154 (99/55)	R	Bulgaria	NSCLC	Pembrolizumab	(1) time to treatment failure < 3 months; (2) increase ≥ 50% in the sum of target lesion major diameters between baseline and first radiological evaluation; (3) appearance of at least two new lesions in an organ already involved between baseline and first radiological evaluation; (4) spread of the disease to a new organ between baseline and first radiological evaluation; and (5) clinical deterioration with ECOG PS ≥ 2 during the first 3 months of treatment. Criteria 1 and 5 were mandatory.	8	8	High	16/154 (10.4%)
Park et al. (29)	2020	125 (103/22)	R	Korea	HNSCC	Anti-PD-1 mAb, anti-PD-L1 mAb, anti-CTLA-4 mAb	TGKpost/TGKpre ≥ 2	15	3	High	18/125 (14.4%)
Sasaki A, et al. (30)	2019	62 (45/17)	R	Japan	Gastric cancer	Nivolumab	TGKpost/TGKpre ≥ 2 > 50% increase in the sum of the total measured tumor burden	12	1	High	13/62 (21%)
Schuiveling M, et al. (31)	2021	162 (99/63)	R	Netherlands	Melanoma	Anti-PD1, anti-CTLA-4	a time to treatment failure less than 2 months with doubling of tumor burden and a two fold increase in tumor growth rate during treatment	2	0	High	2/162 (1.2%)
Yildirim et al. (32)	2022	121 (85/36)	R	Turkey	Multiple cancers	Nivolumab, avelumab, pembrolizumab, atezolizumab	Defined by RECIST progression and at least three of the following symptoms: time to treatment failure <2 months (time to treatment failure is defined as the time from the start of treatment with ICI to ICI discontinuation for any reason); increase of ≥50% in the sum of target lesion major diameters between baseline and first radiologic evaluation; the appearance of at least two new lesions in an organ already involved between baseline and first radiologic evaluation; spread of the disease to a new organ between baseline and first radiologic evaluation; and clinical deterioration with a decrease in ECOG performance status ≥2 during the first 2 months of treatmenta time to treatment failure less than 2 months with doubling of tumor burden and a twofold increase in tumor growth rate during treatment	17	3	High	20/121 (16.5%)
Zhang et al. (33)	2021	69 (57/12)	R	China	HCC	Nivolumab, pembrolizumab, camrelizumab	Between the baseline and the first radiological evaluation; the appearance of at least two new lesions in an organ already involved between baseline and the first radiological evaluation; spread of the disease to a new organ between baseline and the first radiological evaluation; and clinical deterioration with a decrease in the ECOG performance status of ≥ 2 during the first 2 months of treatment (1) TTF < 2 months; (2) disease progression at the first evaluation and a > 50% increase in TGR; (3) TGRpost/TGRpre ≥ 2	9	1	High	10/69 (14.5%)

HPD, hyperprogressive disease; NSCLC, nonsmall cell lung cancer; LC, lung cancer; HCC, hepatocellular carcinoma; GC, gastrointestinal adenocarcinoma; R/M HNSCC, recurrent and/or metastatic head and neck squamous carcinoma; PD-1, programmed cell death protein 1; PD-L1, programmed death 1 ligand 1; CTLA-4, cytotoxic T-lymphocyte antigen 4.

#### 3.1.2 Characteristics of the patients studied

A total of 2,291 cases were included in this study, among which 425 cases (14.5%) were HPD and 1,866 cases were non-HPD. The incidence of HPD in each study ranged from 1.2% to 41.0%. There were 301 male cases and 124 female cases. There were 229 cases of NSCLC, 109 cases of digestive system malignancies, 2 cases of melanoma, 18 cases of head and neck tumors, and the rest were other cancers (**Table 1**).

#### 3.1.3 Characteristics of the design types included in the study

All 20 included studies were retrospective studies (13, 15–29, 32–35). The cases were divided into HPD and non-HPD groups. In assessing tumor growth kinetics indicators, because there is no standard, the definition of HPD varied across the included studies. The criteria reported in the included studies in this analysis were adopted (**Table 1**).

In these studies, Petrova et al. (15) employed criteria that integrated both clinical and radiological parameters. HPD was defined not only as a significant increase in tumor burden but also as the emergence of new lesions in distant organs. Notably, the definition of tumor growth kinetics (TGK) differed in the studies conducted by Kim et al. (24, 26). Furthermore, Zhang et al. (33) introduced two additional requirements in the study by Champiat et al. (11): (1) time to treatment failure (TTF) < 2 months, and (2) disease progression at the first assessment with a tumor growth rate (TGR) increase exceeding 50%.

### 3.2 Association between sex and hyperprogressive disease in cancer patients undergoing immunotherapy

A total of 20 retrospective studies involving 2,291 patients receiving immunotherapy provided numerical data on sex and the incidence of HPD. ORs and 95% CIs from these studies were pooled. In the meta-analysis of gender and HPD (**Figure 2**), no statistically significant difference was observed between men and women (OR = 0.90; 95% CI: 0.75–1.15; *p* = 0.41; *I*^2^ = 27%, *p* = 0.13). Subgroup analyses were conducted based on tumor type, geographic region of included patients, and gender ratio.

**Figure 2:**
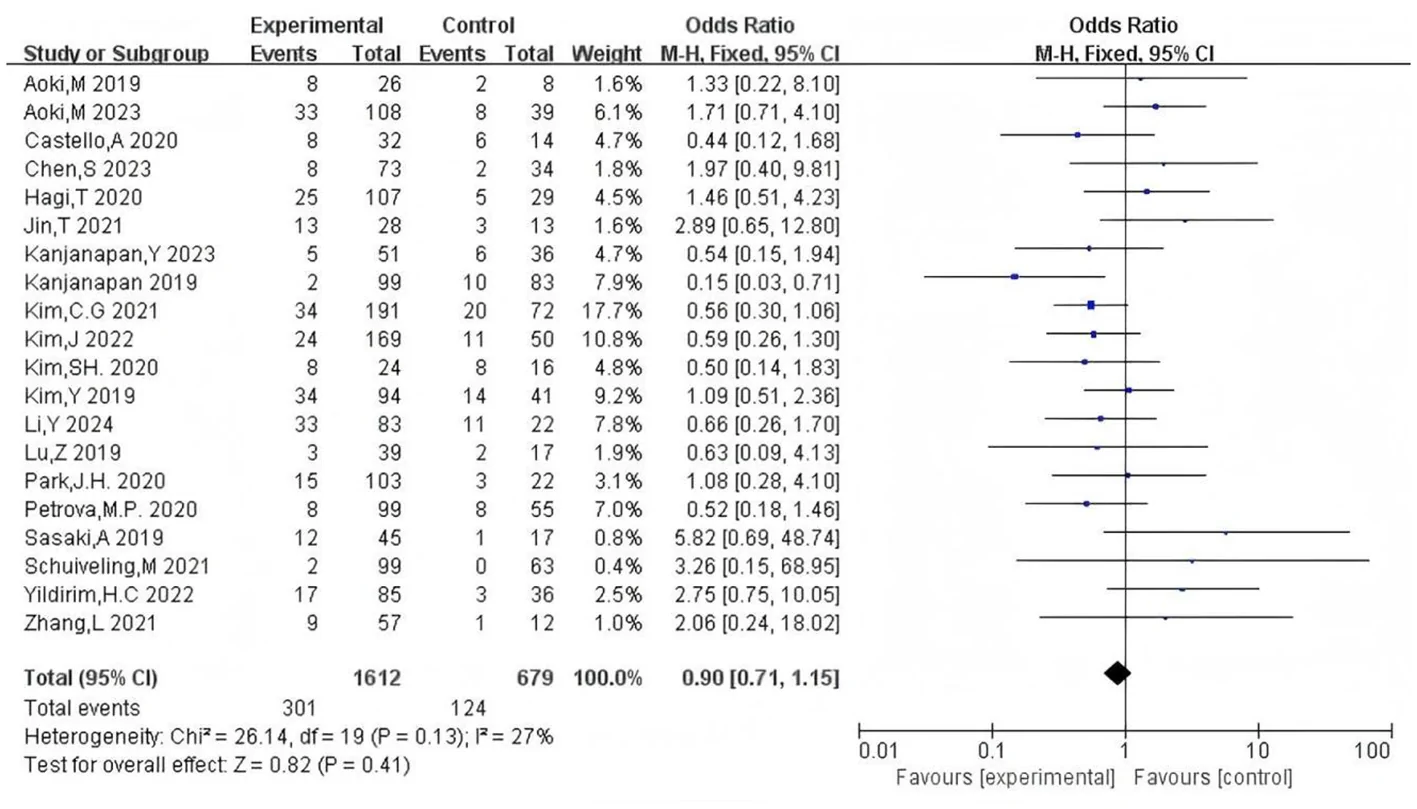
Forest plot of the association between sex and HPD in patients treated with immunotherapy.

### 3.3 Sensitivity analysis and publication bias

The analysis revealed low heterogeneity among the studies (*I*^2^ = 27%; *p* = 0.13). Consequently, a sensitivity analysis was conducted on all included studies. Each study was sequentially excluded from the meta-analysis to evaluate its impact on the pooled OR. The results of the sensitivity analysis demonstrated that all included studies showed no evidence of potential publication bias (**Figure 3**). Furthermore, publication bias was assessed using Begg’s test. Funnel plot assessment (**Supplementary Figure 1**) showed that studies were symmetrically distributed on both sides of the funnel plot, indicating no significant publication bias (*p* = 0.230).

**Figure 3:**
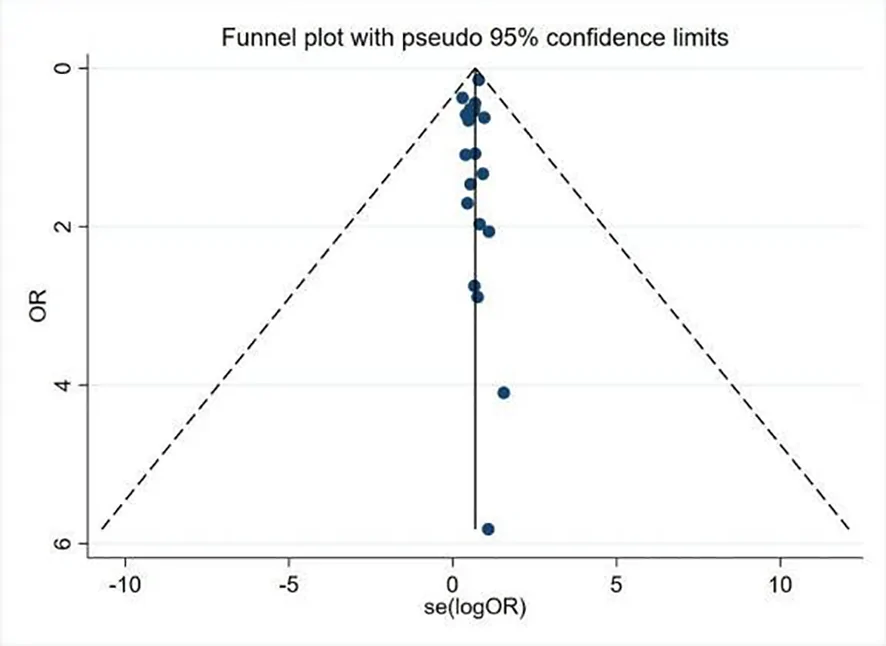
Funnel plot for the association between sex and HPD risk.

### 3.4 Subgroup analysis

The overall analysis demonstrated no significant association between sex and HPD risk (OR = 0.90; 95% CI: 0.71–1. 15; *I*^2^ = 27%; *p* = 0. 13), which supports our core hypothesis that gender should not be considered a determinative factor for HPD risk in ICI therapy. However, the prespecified subgroup analyses revealed several important effect modifiers, including geographic region, tumor type, gender ratio, and treatment modality, all of which warrant further investigation (**Figure 4**). To further investigate the potential impact of heterogeneous HPD diagnostic criteria on the pooled results (**Figure 4**), we performed a subgroup analysis stratifying the included studies according to their HPD definitions. The 20 included studies were categorized into three groups: (1) pure TGR/TGK-based definitions (*n* = 6), (2) pure RECIST 1.1-based definitions (*n* = 2), and (3) composite definitions combining TGR/TGK with TTF and/or new lesions/clinical deterioration (*n* = 12).

**Figure 4:**
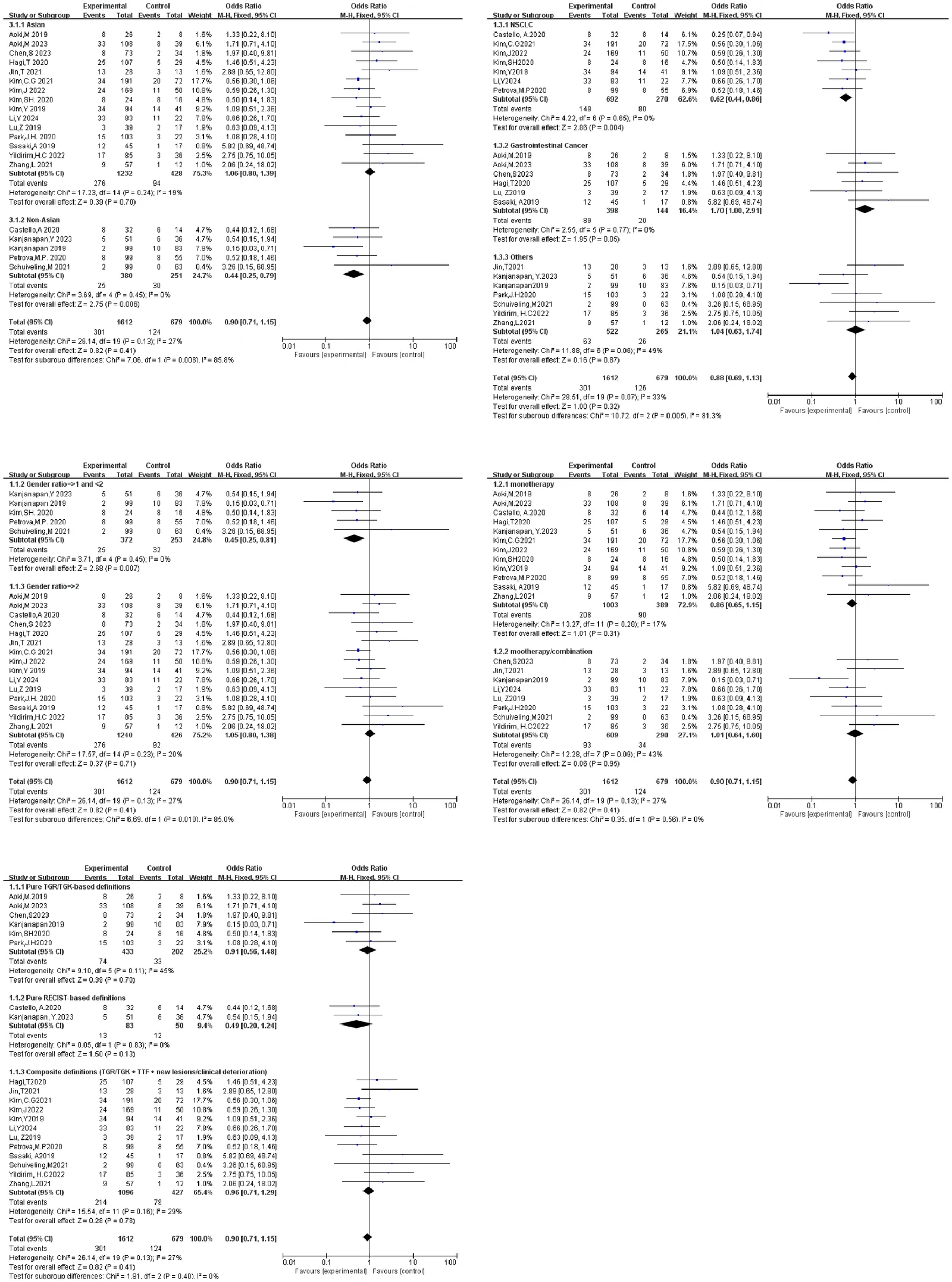
Sex-based subgroup analysis of HPD in immunotherapy patients.

## 4 Discussion

Patients exhibit accelerated disease progression following immunotherapy, a phenomenon characterized by rapid tumor growth rather than control, which has been identified as HPD (23, 34). The incidence of HPD across various studies to date ranges from 4% to 29%, while our meta-analysis reveals an overall HPD incidence spanning from 1.2% to 41.0% (36). The lack of a standardized definition for HPD and its predictive biomarkers remains a challenge. Reported occurrences of HPD during immunotherapy have been shown to significantly reduce patients’ overall survival (37). Therefore, early identification of HPD is of paramount importance. Numerous studies have reported clinical factors that may contribute to a high incidence of HPD, including low PD-L1 expression levels, more than three metastatic sites, elevated NLR, and reduced hemoglobin (Hb) levels (8, 12, 33). In addition to these factors, gender represents a common clinical variable. Kanjanapan’s research identified a higher incidence of HPD among female patients (13). However, our analytical findings revealed no statistically significant association between sex and HPD. Furthermore, across geographical regions and diverse tumor types, we were unable to demonstrate significant gender-based differences in HPD. Multiple factors may account for these observations.

In the context of HPD, sex differences in immune responses may partly explain why men have a higher risk of developing malignant tumors, while women are more prone to autoimmune diseases. Sex disparities may be associated with genetic factors, as female cells contain two X chromosomes, whereas male cells contain one X chromosome and one Y chromosome (7). The latest research by Chen et al. revealed, for the first time, that the loss of the Y chromosome in tumor cells, which is uniquely present in male cells, can facilitate immune evasion by impairing antigen presentation, thereby enabling cancer cells to escape recognition and elimination by the immune system (38). This study elucidates the underlying mechanisms of male predisposition to cancer development and poorer prognosis from the perspective of Y chromosome loss. The research findings demonstrate that the molecular origins of sex disparities in cancer incidence extend beyond hormonal factors, encompassing significant genetic determinants (39). We updated the previous search conducted by Zhao et al. (36) by exclusively including trials that involved immunotherapy and excluding those with insufficient sex-specific data, thereby enabling a focused evaluation of the relationship between sex and HPD in immunotherapy. Although the overall analysis revealed no significant association between sex and HPD risk, significant subgroup differences were observed across tumor types (*p* = 0.005), suggesting that tumor type may modulate the effect of sex on HPD. Our subgroup analysis revealed varying incidence rates of HPD across different sex ratios. Specifically, within a sex ratio range of 1–2, a statistically significant association was observed between patient sex and the occurrence of HPD OR = 0.45 (95% CI: 0.25 - 0.81) *p* < 0.32), with a higher incidence of HPD observed in female patients. In the study by Kanjanapan et al. (13), the sex ratio was between one and two. Notably, the number of studies in the subgroup with a sex ratio greater than two was much larger than that in the subgroup with a sex ratio between one and two, which contributed substantially to the combined HR effect. Furthermore, three studies conducted by Petrova et al. (15), Aoki et al. (16), and Kim et al. (17) employed PD-1/PD-L1 inhibitor monotherapy, while Kanjanapan’s research utilized PD-1/PD-L1 inhibitor monotherapy or combination therapy with CTLA-4 or other ICIs (13). Some studies suggest that the efficacy of combining ICIs with chemotherapy is superior in female patients compared with male patients. This is attributed to chemotherapy’s ability to enhance the tumor mutational burden in women, thereby increasing the antigenicity of cancer cells. Compared with men, women exhibit a more efficient capacity to eliminate tumor cells with higher antigenicity (40). Tumor mutational burden in the study by Kanjanapan et al., as an unadjusted potential confounding factor, may account for the higher incidence of HPD observed in female patients (13). Subgroup analysis according to treatment regimen revealed no significant association between sex and HPD risk in either the monotherapy subgroup (OR = 0.86; 95% CI: 0.65 -1.15; *p* = 0.31; *I*^2^ = 17%) or the combination therapy subgroup (OR = 1.01; 95% CI: 0.64 -1.60; *p* = 0.95; *I*^2^ = 43%). The test for subgroup differences was not significant (*p* = 0.56, *I*^2^ = 0%), indicating that treatment type did not significantly modify the association between sex and HPD risk. Although combination therapy has been hypothesized to potentially amplify sex specific differences in immune responses --particularly through chemotherapy induced immunomodulation or the addition of CTLA-4 inhibitors our findings did not support this hypothesis in the context of HPD risk. Previous meta analyses have shown that immunotherapy plus chemotherapy provides equivalent survival benefits for both sexes across cancer types (HR for OS: 0.76 in males vs. 0.78 in females) (41), suggesting that the addition of chemotherapy does not substantially alter sex-based treatment effects. However, the combination therapy subgroup in our analysis included heterogeneous regimens, such as ICIs combined with chemotherapy or with other ICIs (e.g., CTLA-4 inhibitors), which may have contributed to the wide confidence intervals observed in this subgroup. Furthermore, the combination therapy subgroup had a relatively small sample size and was enriched with specific tumor types (e.g., colorectal cancer, esophageal cancer) that may themselves influence HPD risk. Therefore, the apparent lack of association in the combination therapy subgroup should be interpreted with caution, and future studies with larger sample sizes and homogeneous treatment regimens are needed to further evaluate the potential interaction between sex, treatment type, and HPD risk.

## 5 Limitations

This study has several limitations. First, all 20 included studies were retrospective, which limits causal inference and introduces potential selection and information biases. Second, the marked variability in HPD assessment criteria, tumor growth kinetic indicators, and definitions across studies may impede the pooling of results and potentially introduce selection and reporting biases. This underscores the urgent need for a standardized HPD definition. Third, our analysis relied on unadjusted odds ratios rather than individual patient-level data. We were unable to control for key confounders, including tumor stage, line of immunotherapy, PD-L1 expression, TMB, and menopausal status. The observed effect size may therefore be subject to residual confounding, and our findings should be considered hypothesis-generating rather than confirmatory. Fourth, critical factors such as the menopausal status of female patients, the pivotal role of gender hormones in immune system regulation, and their potential therapeutic implications warrant further consideration in the context of HPD. Finally, future studies should incorporate comprehensive data on gender hormone status and immune-related factors to further elucidate the relationship between sex and HPD risk. Further high-quality, large-scale, multicenter studies are necessary.

## 6 Conclusion

The study concludes that the present meta-analysis does not provide sufficient evidence to support a significant association between patient sex and HPD risk. Subgroup analyses according to geographic region, tumor type, gender ratio, definitions and treatment modality recapitulated these findings.

## Data Availability

The original contributions presented in the study are included in the article/**Supplementary Material**. Further inquiries can be directed to the corresponding author.

## References

[1] Chen H Yang H Guo L Sun Q . The role of immune checkpoint inhibitors in cancer therapy: mechanism and therapeutic advances. Medcomm. (2025) 6:e70412. doi: 10.1002/mco2.70412 41059489 PMC12497686

[2] Frediani JN Fabbri M . Essential role of miRNAs in orchestrating the biology of the tumor microenvironment. Mol Cancer. (2016) 15:42. doi: 10.1186/s12943-016-0525-3 27231010 PMC4882787

[3] Klein SL Flanagan KL . Sex differences in immune responses. Nat Rev Immunol. (2016) 16:626–38. doi: 10.1038/nri.2016.90 27546235

[4] Özdemir BC Csajka C Dotto GP Wagner AD . Sex differences in efficacy and toxicity of systemic treatments: an undervalued issue in the era of precision oncology. J Clin Oncol Oficial J Am Soc Clin Oncol. (2018) 36:2680–3. doi: 10.1038/nri.2016.90 30004815

[5] Cook MB McGlynn KA Devesa SS Freedman ND Anderson WF . Sex disparities in cancer mortality and survival. Cancer Epidemiology Biomarkers Prevention: A Publ Am Assoc For Cancer Research Cosponsored by Am Soc Prev Oncol. (2011) 20:1629–37. doi: 10.1158/1055-9965.Epi-11-0246 21750167 PMC3153584

[6] Zhang Y Liang H Cheng J Choudhry AA Zhou X Zhou G . Associations between sex-specific reproductive factors and risk of new-onset lung cancer among female patients. Chest. (2024) 166:226–39. doi: 10.1016/j.chest.2023.11.014 37977264

[7] Oliva M Muñoz-Aguirre M Kim-Hellmuth S Wucher V Gewirtz ADH Cotter DJ . The impact of sex on gene expression across human tissues. Sci (New York NY). (2020) 369(6509):eaba3066. doi: 10.1126/science.aba3066 32913072 PMC8136152

[8] Zang H Peng J Zheng H Fan S . Hyperprogression after immune-checkpoint inhibitor treatment: characteristics and hypotheses. Front Oncol. (2020) 10:515. doi: 10.3389/fonc.2020.00515 32411591 PMC7201048

[9] Ren Z Yang K Zhu L Yin D Zhou Y . Regulatory T cells as crucial trigger and potential target for hyperprogressive disease subsequent to PD-1/PD-L1 blockade for cancer treatment. Int Immunopharmacol. (2024) 132:111934. doi: 10.1016/j.intimp.2024.111934 38574701

[10] Sun D Qian H Li J Xing P . Targeting MDM2 in Malignancies is a promising strategy for overcoming resistance to anticancer immunotherapy. J BioMed Sci. (2024) 31:17. doi: 10.1186/s12929-024-01004-x 38281981 PMC10823613

[11] Champiat S Dercle L Ammari S Massard C Hollebecque A Postel-Vinay S . Hyperprogressive disease is a new pattern of progression in cancer patients treated by anti-PD-1/PD-L1. Clin Cancer Research: Off J Am Assoc For Cancer Res. (2017) 23:1920–8. doi: 10.1158/1078-0432.Ccr-16-1741 27827313

[12] Montagna DR Duarte A Chiarella P Rearte B Bustuoabad OD Vermeulen M . Inhibition of hyperprogressive cancer disease induced by immune-checkpoint blockade upon co-treatment with meta-tyrosine and p38 pathway inhibitor. BMC Cancer. (2022) 22:845. doi: 10.1186/s12885-022-09941-2 35922755 PMC9347122

[13] Kanjanapan Y Day D Wang L Al‐Sawaihey H Abbas E Namini A . Hyperprogressive disease in early‐phase immunotherapy trials: clinical predictors and association with immune‐related toxicities. Cancer. (2019) 125:1341–9. doi: 10.1002/cncr.31999 30768786

[14] Saâda-Bouzid E Defaucheux C Karabajakian A Coloma VP Servois V Paoletti X . Hyperprogression during anti-PD-1/PD-L1 therapy in patients with recurrent and/or metastatic head and neck squamous cell carcinoma. Ann Oncol Off J Eur Soc For Med Oncol. (2017) 28:1605–11. doi: 10.1093/annonc/mdx178 28419181

[15] Petrova MP Donev IS Radanova MA Eneva MI Dimitrova EG Valchev GN . Sarcopenia and high NLR are associated with the development of hyperprogressive disease after second-line pembrolizumab in patients with non-small-cell lung cancer. Clin Exp Immunol. (2020) 202:353–62. doi: 10.1111/cei.13505 32757277 PMC7670147

[16] Aoki M Kadowaki S Takahashi N Suzuki T Oshima K Ando T . Pattern of disease progression during third-line or later chemotherapy with nivolumab associated with poor prognosis in advanced gastric cancer: a multicenter retrospective study in Japan. Gastric Cancer. (2022) 26:132–44. doi: 10.1007/s10120-022-01349-y 36316527 PMC9813080

[17] Kim J Kim T Jang TW Kang H Kim MH Yoon SH . Clinical outcomes of hyperprogression based on volumetry in non‐small cell lung cancer after immune checkpoint inhibitor treatment. Thorac Cancer. (2022) 13:2170–9. doi: 10.1111/1759-7714.14539 35785522 PMC9346184

[18] Aoki M Shoji H Nagashima K Imazeki H Miyamoto T Hirano H . Hyperprogressive disease during nivolumab or irinotecan treatment in patients with advanced gastric cancer. ESMO Open. (2019) 4(3):e000488. doi: 10.1136/esmoopen-2019-000488 31231567 PMC6555603

[19] Castello A Rossi S Mazziotti E Toschi L Lopci E . Hyperprogressive disease in patients with non–small cell lung cancer treated with checkpoint inhibitors: the role of 18F-FDG PET/CT. J Nucl Med. (2020) 61:821–6. doi: 10.2967/jnumed.119.237768 31862803

[20] Chen S Han L Guo S Tan Z Dai G . Hyperprogressive disease during PD-1 blockade in patients with advanced pancreatic cancer. Hum Vaccines Immunotherapeutics. (2023) 19(2):2252692. doi: 10.1080/21645515.2023.2252692 37675466 PMC10486295

[21] Hagi T Kurokawa Y Kawabata R Omori T Matsuyama J Fujitani K . Multicentre biomarker cohort study on the efficacy of nivolumab treatment for gastric cancer. Br J Cancer. (2020) 123:965–72. doi: 10.1038/s41416-020-0975-7 32616848 PMC7492241

[22] Jin T Zhang Q Jin Q-F Hua Y-H Chen X-Z . Anti-PD1 checkpoint inhibitor with or without chemotherapy for patients with recurrent and metastatic nasopharyngeal carcinoma. Transl Oncol. (2021) 14(2):100989. doi: 10.1016/j.tranon.2020.100989 33341487 PMC7750692

[23] Kanjanapan Y Guduguntla G Varikara AK Szajer J Yip D Cockburn J . Hyperprogressive disease (HPD) in solid tumours receiving immune checkpoint inhibitors in a real-world setting. Technol Cancer Res Treat. (2023) 22:15330338231209129. doi: 10.1177/15330338231209129 37885403 PMC10612441

[24] Kim CG Kim KH Pyo KH Xin CF Hong MH Ahn BC . Hyperprogressive disease during PD-1/PD-L1 blockade in patients with non-small-cell lung cancer. Ann Oncol. (2019) 30:1104–13. doi: 10.1093/annonc/mdz123 30977778

[25] Kim SH Choi C-M Lee DH Kim S-W Yoon S Kim WS . Clinical outcomes of nivolumab in patients with advanced non-small cell lung cancer in real-world practice, with an emphasis on hyper-progressive disease. J Cancer Res Clin Oncol. (2020) 146:3025–36. doi: 10.1007/s00432-020-03293-9 32583235 PMC11804452

[26] Kim Y Kim CH Lee HY Lee S-H Kim HS Lee S . Comprehensive clinical and genetic characterization of hyperprogression based on volumetry in advanced non–small cell lung cancer treated with immune checkpoint inhibitor. J Thorac Oncol. (2019) 14:1608–18. doi: 10.1016/j.jtho.2019.05.033 31195179

[27] Lu Z Zou J Hu Y Li S Zhou T Gong J . Serological markers associated with response to immune checkpoint blockade in metastatic gastrointestinal tract cancer. JAMA Netw Open. (2019) 2(7):e197621. doi: 10.1001/jamanetworkopen.2019.7621 31339548 PMC6659353

[28] Li Y Wang P Xu J Shi X Yin T Teng F . Noninvasive radiomic biomarkers for predicting pseudoprogression and hyperprogression in patients with non-small cell lung cancer treated with immune checkpoint inhibition. Oncoimmunology. (2024) 13:2312628. doi: 10.1080/2162402x.2024.2312628 38343749 PMC10857548

[29] Park HJ Kim KW Won SE Yoon S Chae YK Tirumani SH . Definition, incidence, and challenges for assessment of hyperprogressive disease during cancer treatment with immune checkpoint inhibitors. JAMA Netw Open. (2021) 4(3):e211136. doi: 10.1001/jamanetworkopen.2021.1136 33760090 PMC7991969

[30] Sasaki A Nakamura Y Mishima S Kawazoe A Kuboki Y Bando H . Predictive factors for hyperprogressive disease during nivolumab as anti-PD1 treatment in patients with advanced gastric cancer. Gastric Cancer (2019) 22(4):793–802. 10.1007/s10120-018-00922-8 30627987

[31] Schuiveling M Tonk EHJ Verheijden RJ Suijkerbuijk KPM . Hyperprogressive disease rarely occurs during checkpoint inhibitor treatment for advanced melanoma. Cancer Immunology, Immunotherapy (2020) 70(5):1491–96. 10.1007/s00262-020-02716-3 PMC8053186 32929554

[32] Yildirim HC Guven DC Aktepe OH Taban H Yilmaz F Yasar S . Blood based biomarkers as predictive factors for hyperprogressive disease. J Clin Med. (2022) 11(17):5171. doi: 10.3390/jcm11175171 36079101 PMC9457309

[33] Zhang L Wu L Chen Q Zhang B Liu J Liu S . Predicting hyperprogressive disease in patients with advanced hepatocellular carcinoma treated with anti-programmed cell death 1 therapy. EClinicalMedicine. (2020) 31:100673. doi: 10.1016/j.eclinm.2020.100673 33554079 PMC7846667

[34] Hwang I Park I Yoon SK Lee JL . Hyperprogressive disease in patients with urothelial carcinoma or renal cell carcinoma treated with PD-1/PD-L1 inhibitors. Clin Genitourin Cancer. (2020) 18:e122–33. doi: 10.1016/j.clgc.2019.09.009 31837940

[35] Kamada T Togashi Y Tay C Ha D Sasaki A Nakamura Y . PD-1(+) regulatory T cells amplified by PD-1 blockade promote hyperprogression of cancer. PNAS. (2019) 116:9999–10008. doi: 10.1073/pnas.1822001116 31028147 PMC6525547

[36] Zhao Z Bian J Zhang J Zhang T Lu X . Hyperprogressive disease in patients suffering from solid Malignancies treated by immune checkpoint inhibitors: a systematic review and meta-analysis. Front Oncol. (2022) 12. doi: 10.3389/fonc.2022.843707 35992878 PMC9381837

[37] Choi YJ Kim T Kim EY Lee SH Kwon DS Chang YS . Prediction model for hyperprogressive disease in non-small cell lung cancer treated with immune checkpoint inhibitors. Thorac Cancer. (2020) 11:2793–803. doi: 10.1111/1759-7714.13594 32779394 PMC7529559

[38] Chen X Shen Y Choi S Abdel-Hafiz HA Basu M Hoelzen L . Concurrent loss of the Y chromosome in cancer and T cells impacts outcome. Nature. (2025) 642(8069):1041–50. doi: 10.1038/s41586-025-09071-2 40468066 PMC12221978

[39] Abdel-Hafiz HA Schafer JM Chen X Xiao T Gauntner TD Li Z . Y chromosome loss in cancer drives growth by evasion of adaptive immunity. Nature. (2023) 619:624–31. doi: 10.1038/s41586-023-06234-x 37344596 PMC10975863

[40] Liang J Hong J Tang X Qiu X Zhu K Zhou L . Sex difference in response to non-small cell lung cancer immunotherapy: an updated meta-analysis. Ann Med. (2022) 54:2606–16. doi: 10.1080/07853890.2022.2124449 36128737 PMC9518269

[41] Abdu SA Asaad WA Wdaan M Li N . Sex differences in immunotherapy plus chemotherapy: A systematic review and meta-analysis. Crit Rev Oncol Hematol. (2026) 218:105069. doi: 10.1016/j.critrevonc.2025.105069 41360348

