# Supplementary material for: Sex and risk of hyperprogressive disease in cancer patients receiving immune checkpoint inhibitors: a systematic review and meta-analysis
Source: Front Immunol. 2026 Sep 16;17:1860831. doi: 10.3389/fimmu.2026.1860831 (PMC13623834; doi:10.3389/fimmu.2026.1860831)
Supplement: Supplementary file 1 [file Table1.docx]

Search: (((gender[Title/Abstract]) OR (sex[Title/Abstract])) OR (sexuality[Title/Abstract])) AND ((((((hyperprogression[Title/Abstract]) OR (hyperprogressive disease[Title/Abstract])) OR (HPD[Title/Abstract])) AND ((((((((programmed cell death-1 inhibitor) OR (PD-1 inhibitor, programmed cell death-ligand 1 inhibitor)) OR (PD-L1 inhibitor, cytotoxic T lymphocyte-associated antigen-4 inhibitor)) OR (CTLA-4 inhibitor)) OR (ipilimumab)) OR (nivolumab)) OR (immune checkpoint inhibitor)) OR (immunotherapy))) AND (((cancer) OR (carcinoma)) OR (tumor)))
